# Multiplex PCR for simultaneous identification of the most common European Opisthorchiid and Heterophyid in fish or fish products

**DOI:** 10.1016/j.fawpar.2020.e00081

**Published:** 2020-05-11

**Authors:** Monica Caffara, Andrea Gustinelli, Angelica Mazzone, Maria L. Fioravanti

**Affiliations:** Department of Veterinary Medical Sciences, Alma Mater Studiorum University of Bologna, Via Tolara di Sopra 50, 40064 Ozzano Emilia (BO), Italy

**Keywords:** Multiplex PCR, Opisthorchiidae, Heterophyidae, Freshwater fish, Fish products, Europe

## Abstract

Among others, the families Opisthorchiidae and Heterophyidae includes several genera causing fish-borne zoonoses and distributed also in European Countries and that are included in the ParaFishControl (Advanced Tools and Research Strategies for Parasite Control in European farmed fish) H2020 EU project. Due to the small size of the metacercariae, the infective stage for human, these parasites cannot be detected visually in fish and monitoring requires expert application of time-consuming techniques. The aim of this was to develop a rapid and affordable molecular method based on multiplex PCR for simultaneous identification of metacercariae of the most common European Opisthorchiid and Heterophyid in fish or fish products.

## 1 Introduction

Fish-borne zoonotic trematodes are commonly reported worldwide, and >70 species are known to be zoonotic (WHO, 2011). Among others, the families Opisthorchiidae and Heterophyidae include several genera of zoonotic interest distributed also in European Countries. The life cycle of these parasites involves a snail as first intermediate host, fish as second intermediate host, mammals, including man, and fish-eating birds as definitive hosts (Chai et al., 2007). Humans and other definitive hosts can become infected by eating raw or undercooked fish or fish product containing infective metacercariae (Chai et al., 2007; Phan et al., 2011).

Members of the family Opisthorchiidae mature mainly in the hepatobiliary system of many piscivorous mammals and birds (Lim et al., 2008; Marcos et al., 2008). It includes 33 genera (King and Scholz, 2001) among which 3 species belonging to 2 genera are of major zoonotic importance such as *Opisthorchis felineus* (Rivolta, 1884), *O. viverrini* (Poirier, 1886) and *Clonorchis sinensis* (Cobbold, 1875) (the last two not present in Europe) and some considered as minor zoonotic agents, *Metorchis* spp. (*M. bilis* Braun, 1890) and *Pseudamphistomum truncatum* (Rudolphi, 1819), both described in Eurasia (Sherrard-Smith et al., 2009; Mordvinov et al., 2012). The family Heterophyidae includes trematodes infecting vertebrate animals, including mammals and birds. At least 36 genera are known within this family (Pearson, 2008), with at least 13 genera and 29 species known to be zoonotic (Chai and Jung, 2017), among which *Metagonimus* spp. and *Apophallus* spp. have been reported in Europe.

Transmission to humans occurs through the consumption of raw or undercooked fish muscle infected by the larval stage of the parasite, the metacercaria. Since the morphological approach poses some issues in identifying the species at this stage and discriminating zoonotic and non-zoonotic metacercariae, molecular techniques are necessary for their identification.

Due to the small size of the metacercariae (generally <400 μm), these parasites cannot be visually detected in fish and monitoring requires expert application of time-consuming techniques. The standard procedure, reported by the European Union Reference Laboratory for Parasites (ISS, Rome) to detect opisthorchiid/heterophyid metacercariae in fish fillets primarily involves artificial digestion of fish tissue with hydrochloric-pepsin or the compression of muscle between two-glass slides and observation under a stereomicroscope. Isolated metacercariae are then identified to genus level using morphological features, but they are very often difficult to distinguish due to the high morphological similarity.

In the framework of the EU project ParaFishControl (H2020, Advanced Tools and Research Strategies for Parasite Control in European farmed fish) the aim of this work was to develop a rapid and affordable molecular method for simultaneous identification of metacercariae of the most common European opisthorchiid and heterophyid in fish or fish products.

## 2 Materials and methods

For the design of the multiplex PCR, cercariae or metacercariae of opisthorchiid/heterophyid were collected. In details, opisthorchiid metacercariae of *Opisthorchis felineus* were isolated from the muscle of wild tench (*Tinca tinca*) from Bolsena Lake, central Italy and *Pseudamphistomum truncatum* metacercariae from wild tench sampled at Como Lake, northern Italy, while *Metorchis* spp. cercariae were collected from gastropods sampled from Iseo Lake, northern Italy. Heterophyid metacercariae of *Metagonimus* spp. were collected from *Alburnus alburnus* and *Chondrostoma nasus* while metacercariae of *Apophallus* spp., from *C. nasus*, *Abramis brama*, *Blicca bjoerkna*, *Gymnocephalus cernua*, *Perca fluviatilis*, *Scardinius erythopthalmus* and *Esox lucius* sampled in Hungary.

From all the developmental stages the DNA was extracted using Chelex100 (Sigma-Aldrich, Saint Louis, MO, USA) at 5% concentration. Briefly, 300 μl of 5% Chelex100 were added to 1 cercaria or metacercaria, incubated in heat block at 95 °C for 5 min. and centrifuged at full speed for 5 min. We run incubations from 5 to 20 min. and because we observed no differences, selected the shortest one. The supernatant containing the DNA was transferred into a clean tube and diluted at least at 1:10 for downstream use.

Three molecular markers were selected, 18S and ITS rDNA, and COI mtDNA and amplified by PCR with the primers reported in Table 1. The products were resolved on a 1% agarose gel stained with SYBR Safe DNA Gel Stain in 0.5× TBE (Invitrogen – Thermo Fisher Scientific, Carlsbad, CA, USA). For sequencing, bands were excised and purified by NucleoSpin Gel and PCR Cleanup (Mackerey-Nagel, Düren, Germany) and sequenced in both direction with an ABI 3730 DNA analyzer at StarSEQ GmbH (Mainz, Germany). The sequences were assembled with Vector NTI Advance™ 11 software (Thermo Fisher Scientific, Carlsbad, CA, USA). In order to confirm their identity, the sequence of each molecular marker was firstly subjected to BLAST search and then aligned using ClustalW included in Bioedit software and adjusted by eye.

**Table 1:** Primers used for the amplifications of the 18S and ITS rDNA and COI mtDNA.

	Gene	References
82_f - CAGTAGTCATATGCTTGTCTCAG	18S rRNA	Mariaux, 1998
81_r - TTCACCTACGGAAACCTTGTTACG		
83_f - GATACCGTCCTAGTTCTGACCA		
84_r - TCCTTTAAGTTTCAGCTT GC		
81_f - GTAACAAGGTTTCCGTAGGTGAA	ITS rRNA	Gustinelli et al., 2010
ITS2_r - CCTGGTTAGTTTCTTTTCCTCCGC		Cribb et al., 1998
Dice1F ATTAACCCTCACTAAATTWCNTTRGATCATAAG^a^	COI mtDNA	Van Steenkiste et al., 2015
Dice11R TAATACGACTCACTATAGCWGWACHAAATTTHCGATC		

a Shortened T3 and T7 tails at the 5′ end of Dice1F and Dice11R, are underlined and were used for sequencing.

Multiplex PCR primers were designed manually or using Primer-BLAST and then checked for primer-dimers with the web tool Multiple Primers Analyzer (Thermo Fisher Scientific). The primers were chosen basing on different fragments size in order to distinguish *O. felineus*, *P. truncatum*, *Metorchis* spp., *Apophallus* spp. and *Metagonimus* spp.

## 3 Results

All the molecular markers for all the five genera/species under consideration were successfully amplified and sequenced.

The sequences of the 18S rDNA gene was ~1860 bp; however, the multiple alignment showed no possibility to find a regions variable enough which could be used to design primers, as it resulted to be highly conserved (overall distance 0.01). For this reason, it was not further considered. The sequences of the entire ITS rDNA was ~1600 bp (including the end of 18S and the beginning of 28S). The multiple alignment showed the presence of variable regions in which some of the primers were designed. A common reverse primer (comm_r) was designed at the beginning of the 28S rDNA, and genus specific primers were designed for *Apophallus* spp. (~1066 bp) and for *Metagonimus* spp. (~722 bp). The ITS rDNA primer set yielded multiple amplifications among genera in *O. felineus*, *Metorchis* spp. and *P. truncatum*, and a COI mtDNA primer set was therefore developed. The length of the COI mtDNA sequence was ~640 bp. In this gene the high variability allowed design of primers for *Metorchis* spp. (~500 bp) and *P. truncatum* (~150 bp). Finally, for *O. felineus* the species-specific primers designed previously by Caffara et al. (2017) were used (~230 bp). In Table 2 the list of the final set of primers designed and tested is reported.

**Table 2:** Primers designed and tested in the present study.

		Gene	Product
Ap4_f	CCAAGCGAAATCTTCCAAGG	ITS rRNA	~1066 bp
Mt3_f	TTGAATAATGTGACACGAGC		~722 bp
comm_r	ATATGCTTAAGTTCAGCGGG		
Me4_f	GTGGGTTTTTAGGACTTGGG	COI mtDNA	~500 bp
Me4_r	ACCTGCTGCCAACACAGGTAA		
Ps1_f	CCTCTCCTGTTAGGGTTGTC		~150 bp
Ps1_r	CCCGATGACAGGGGAGGATA		
OF9_f	TGTGAGGCGGGTTTACAGGA		~230 bp (Caffara et al., 2017)
OF6_r	CTCCAGCCCCACCATACATT		

Ap = *Apophallus*, Mt = *Metagonimus*, Me = *Metorchis*, Ps = *Pseudamphistomum*, OF = *Opisthorchis felineus*, comm_r = common reverse.

The primer set was tested firstly in singlet PCR of 25 μl, with 2× DreamTaq Hot Start Green PCR Master Mix (Thermo Scientific, Thermo Fisher Scientific, Carlsbad, CA, USA) containing 2× DreamTaq buffer, dNTP's (0.4 mM of each), 4 mM MgCl_2_, 0.3 μM of each primer and 2.5 μl of template DNA. The thermal cycler program (Tpersonal, Biometra) was 40 cycles of 30 s at 95 °C, 30 s at 50 °C and 1 min at 72 °C, preceded by a denaturation step at 95 °C for 3 min and followed by an extended elongation step at 72 °C for 10 min. The products were resolved in 1% agarose gel stained with SYBR Safe DNA Gel Stain in 0.5× TBE. All the primer sets successfully amplified products of the expected size.

**Fig. 1:**
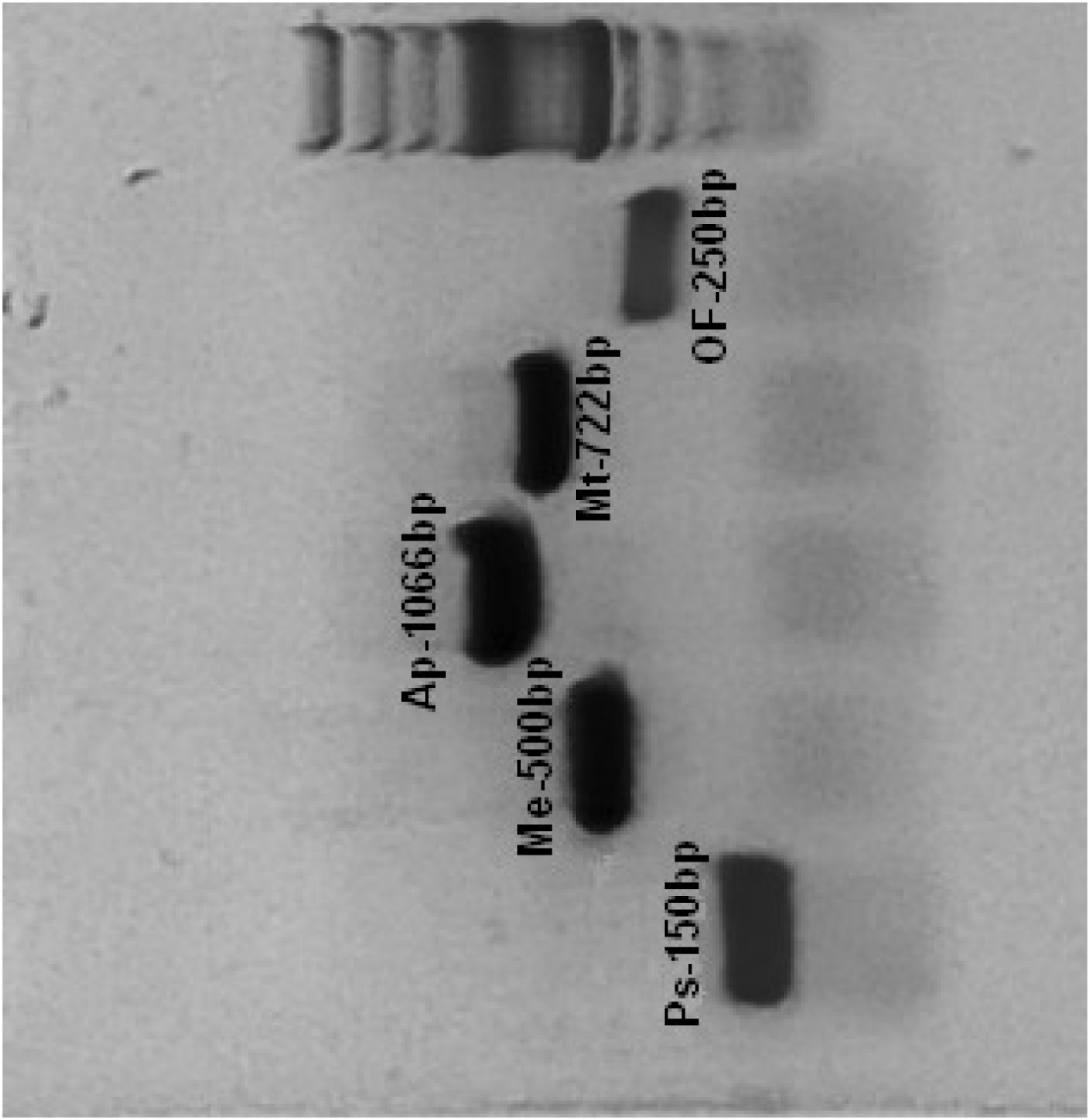
Multiplex-PCR products of *Pseudamphistomum truncatum* (Ps), *Metorchis* spp. (Me), *Apophallus* spp. (Ap), *Metagonimus* spp. (Mt) and *Opisthorchis felineus* (OF). Molecular marker 100 bp.

The primers were then combined in multiplex PCR using the same protocol as singlet PCR. Different primers concentrations were tested: 0.25 μM, 0.2 μM and 0.1 μM. The concentration giving the best results was 0.2 μM. The combined primers successfully amplified the expected products, even if some aspecific bands were present. To evaluate the specificity of these primer set and the conditions of aspecific binding, a PCR gradient using Veriti® 96-Well Thermal Cycler (Applied Biosystem) was performed twice. The annealing temperatures tested were from 51 °C to 56 °C and from 57 °C to 62 °C. An annealing temperature of 60 °C gave the highest specific band intensity without non-specific products (Fig. 1). Finally, all the genus/species specific bands obtained were excised, purified and sequenced (see Materials and Methods) in order to confirm their belonging to the genus/species. The BLAST results confirmed the identity of *O. felineus* (EU921260)*, P. truncatum* (KP869085), *Metorchis* spp. (KY075779), *Metagonimus* spp. (MF407173) and *Apophallus* spp. (MF438057 *A. donicus* and MF438066 *A. muehlingi*).

## 4 Discussion

A central prerequisite for control of food-borne zoonotic trematodes is an accurate diagnosis at least at genus level. Freshwater fish muscles can harbor metacercariae of species that differ in zoonotic importance and that cannot be distinguished with conventional light microscopy. The multiplex PCR developed in the present study may provide a rapid and cost-effective tool for the simultaneous identification of *O. felineus*, *P. truncatum*, *Metorchis* spp., *Metagonimus* spp. and *Apophallus* spp. the main zoonotic or potentially zoonotic trematodes present in Europe. Moreover, this multiplex PCR could be used to identify the abovementioned genus/species at any developmental stages (eggs, cercariae, metacercariae and adult). Currently, the identification of these genera/species is based on PCR followed by sequencing, a time-consuming and expensive technique, except for *O. felineus* for which a species-specific primer has been published previously by Caffara et al. (2017). Multiplex PCR has been successfully developed for other zoonotic parasites or their hosts, such as for example *C. sinensis* and *O. viverrini* (Le et al., 2006), for distinguish among *Galba* species, host of *Fasciola hepatica* (Alda et al., 2018), or simultaneous detection of *Schistosoma mansoni* and the *Biomphalaria* (Jannotti-Passos et al., 2006). In conclusion the multiplex PCR herein developed is an accurate and affordable method that avoids the costs of sequencing and enables rapid identification of any developmental stage of five potentially zoonotic agents in a single reaction.

## Funding

This work was funded by the 10.13039/501100000780European Union, through the 10.13039/100010661Horizon 2020 - Research and Innovation Framework Programme grant agreement 634429 (ParaFishControl).

## Declaration of competing interest

The authors declare that they have no known competing financial interests or personal relationships that could have appeared to influence the work reported in this paper.
